# New Approach against Chondrosoma Cells—Cold Plasma Treatment Inhibits Cell Motility and Metabolism, and Leads to Apoptosis

**DOI:** 10.3390/biomedicines10030688

**Published:** 2022-03-17

**Authors:** Andreas Nitsch, Silas Strakeljahn, Josephine M. Jacoby, Konrad F. Sieb, Alexander Mustea, Sander Bekeschus, Axel Ekkernkamp, Matthias B. Stope, Lyubomir Haralambiev

**Affiliations:** 1 Center for Orthopaedics, Trauma Surgery and Rehabilitation Medicine, University Medicine Greifswald, Ferdinand-Sauerbruch-Straße, 17475 Greifswald, Germany; andreas.nitsch@med.uni-greifswald.de (A.N.); ss166320@uni-greifswald.de (S.S.); josephinemarie.jacoby@stud.uni-greifswald.de (J.M.J.); s-kosieb@uni-greifswald.de (K.F.S.); ekkernkamp@ukb.de (A.E.); 2 Department of Gynecology and Gynecological Oncology, University Hospital Bonn, Venusberg-Campus 1, 53127 Bonn, Germany; alexander.mustea@ukbonn.de (A.M.); matthias.stope@ukbonn.de (M.B.S.); 3 ZIK Plasmatis, Leibniz Institute for Plasma Science and Technology (INP), Felix-Hausdorff-Str. 2, 17489 Greifswald, Germany; sander.bekeschus@inp-greifswald.de; 4 Department of Trauma and Orthopaedic Surgery, BG Klinikum Unfallkrankenhaus Berlin, Warener Straße 7, 12683 Berlin, Germany

**Keywords:** SW 1353, CAL 78, cold atmospheric pressure plasma, live/dead cell imaging, radius cell migration assay, caspase-3/7 assay, TUNEL assay

## Abstract

(1) Background: Chondrosarcoma (CS) is a malignant primary bone tumor with a cartilaginous origin. Its slow cell division and severely restricted vascularization are responsible for its poor responsiveness to chemotherapy and radiotherapy. The decisive factor for the prognosis of CS patients is the only adequate therapy—surgical resection. Cold atmospheric pressure plasma (CAP) is emerging as a new option in anti-cancer therapy. Its effect on chondrosarcomas has been poorly investigated. (2) Methods: Two CS cell lines—SW 1353 and CAL 78—were used. Various assays, such as cell growth kinetics, glucose uptake, and metabolic activity assay, along with two different apoptosis assays were performed after CAP treatment. A radius cell migration assay was used to examine cell motility. (3) Results: Both cell lines showed different growth behavior, which was taken into account when using the assays. After CAP treatment, a reduction in metabolic activity was observed in both cell lines. The immediate effect of CAP showed a reduction in cell numbers and in influence on this cell line’s growth rate. The measurement of the glucose concentration in the cell culture medium showed an increase after CAP treatment. Live-dead cell imaging shows an increase in the proportion of dead cells over the incubation time for both cell lines. There was a significant increase in apoptotic signals after 48 h and 72 h for both cell lines in both assays. The migration assay showed that CAP treatment inhibited the motility of chondrosarcoma cells. The effects in all experiments were related to the duration of CAP exposure. (4) Conclusions: The CAP treatment of CS cells inhibits their growth, motility, and metabolism by initiating apoptotic processes.

## 1. Introduction

Chondrosarcoma (CS) is the second most common malignant primary tumor in the human skeleton after osteosarcoma, with an annual incidence of about 3 new cases per million [1,2,3]. It predominantly affects adults over the age of 60 and is characterized by the formation of hyaline cartilaginous, slow-growing, neoplastic tissue [2,4,5]. In the 2020 WHO classification, CS is classified into 8 subtypes: (1) central atypical cartilaginous tumor (ACT)/chondrosarcoma, grade 1 (CS1); (2) secondary peripheral ACT/CS1; (3) central chondrosarcoma, grades 2 and 3; (4) secondary peripheral chondrosarcoma, grades 2 and 3; (5) periosteal chondrosarcoma; (6) clear cell chondrosarcoma; (7) mesenchymal chondrosarcoma; (8) dedifferentiated chondrosarcoma [4]. Primary CS occurs without any correlation to previous bone disease, while secondary CS develops based on pre-existing benign cartilage tumors, such as enchondroma or osteochondroma. Secondary CS occurs both centrally and peripherally in the bone. The typical feature of primary CS is its first manifestation, which is always centrally located [5]. The potential for metastasis from CS is 5–10%. This may be due to the difficult access to the slowly dividing CS cells, caused by the poor vascularization of the hyaline cartilage matrix [2,6]. These properties are also linked to CS’s poor response to chemotherapy and radiation therapy [3,7]. In addition, the expression of certain genes in CS cells, such as the multidrug resistance 1 gene and P-glycoprotein, confers resistance to chemotherapy [8]. Thus, the prognosis for CS patients depends, on the one hand, on histological classification, and on the other hand, on the adequate treatment of the tumor [2]. Since the responsiveness to chemotherapy and radiotherapy of CS is extremely low, the only curative option left is surgical CS resection. The extent of the surgical intervention depends on the stage and extent of the CS. High-grade CS lesions often require radical en bloc excision, while thorough intralesional curettage is sufficient for low-grade CS [9,10]. 

In the search for additional anti-oncological therapy options, cold atmospheric pressure plasma (CAP) has been considered for numerous types of tumors [11,12,13,14]. Its medicinal use is facilitated by its operation at body temperature. Mechanistically, the biomedical effects of CAP are mediated by the generation of various reactive oxygen and nitrogen species (*ROS/RNS*) [15]. Previous studies have detected apoptosis induction, inhibition of growth, and changes in cell membrane integrity as a result of CAP treatment on osteosarcoma and Ewing’s sarcoma cell lines [16,17]. However, the effect of CAP in the area of chondrosarcomas has been poorly investigated to date and has only been described by our working group on the cell membrane [18]. In order to understand the effects of CAP on CS in detail, further fundamental investigations are necessary, which are dealt with in this work.

## 2. Materials and Methods

### 2.1. Cell Culture

The human chondrosarcoma cell lines, CAL-78 (Leibniz Institute DSMZ-German Collection of Microorganisms and Cell Cultures, Braunschweig, Germany) and SW1353 (American Type Culture Collection, Manassas, VA, USA), were propagated at 37 °C and 5% CO_2_. For CAL-78, RPMI 1640 media was supplemented with 1% penicillin/streptomycin (P/S) and 20% fetal calf serum (FCS) and used. SW1353 cells were propagated in Dulbecco’s modified Eagle’s medium (DMEM)/F12 containing stable glutamine, 1.2 g/L NaHCO_3_, 10% FCS, and 1% P/S (all reagents from PAN Biotech, Aidenbach, Germany). 

### 2.2. Calculation of Growth Rate and Doubling Time

For this calculation, 1.0 × 10^4^ cells were propagated in 24-well cell culture plates for 11 days (CAL-78) or 5 days (SW1353) at 37 °C and 5% CO_2_. Viable cells were counted after every 24 h with the CASY cell counter and analyzer model TT (OLS OMNI Life Science, Bremen, Germany). To discriminate against cell debris, dead cells and living cells, gates of 7.20 μm/14.85 μm (CAL-78) and 7.20 μm/13.95 μm (SW1353) were used.

Growth rates were calculated using the following standard exponential growth model provided by GraphPad Prism [19]:

(1) Y=Y0·exp(k·X)

where *Y* represents the cells, *Y0* represents the cell count at *t* = 0, *k* represents the growth rate, and *t* = time.

*Doubling time* was calculated from the growth rate using the following formula:

(2) $$doubling\;time=\frac{\ln\left(2\right)}{k}$$

### 2.3. Metabolic Activity Assay after CAP-Treatment

To test metabolic activity, 1.0 × 10^4^ cells were suspended in 200 µL of the medium and treated with kINPen med (Neoplas tools, Greifswald, Germany) for 5 s, 10 s, and 20 s with CAP or the carrier gas argon. After 72 h of incubation, 40 µL of CellTiter-Blue were added to each well and incubated for 2 h. Fluorescence was recorded at 590 nm using a microplate reader (TECAN Infinite m200, Männedorf, Switzerland). Blanks were subtracted, and the fluorescence of CAP-treated cells was normalized to the corresponding argon control.

### 2.4. Growth Kinetics after CAP Treatment

For testing growth kinetics, 1.0 × 10^4^ cells were suspended in 200 μL culture media and treated with CAP or carrier gas argon for 5 s, 10 s, and 20 s. Next, 800 μL culture media were added, and cells were incubated at 5% CO_2_ and 37 °C. Viable cells were counted after 4 h and then every 24 h, over an incubation period of 9 days (CAL-78) and 5 days (SW1353), with a CASY cell counter and analyzer. The growth rate and the number of cells at *t* = 0 were calculated using the model described in Section 2.2.

### 2.5. Glucose Assay 

For the glucose assay, 200 μL ultrapure water containing 400 mg/L glucose was treated with CAP or carrier gas argon for 5 s, 10 s, and 20 s. Glucose concentration was determined with an enzymatic glucose detection kit (R-Biopharm, Pfungstadt, Germany). Absorption was recorded at 340 nm using a microplate reader (Tecan m200, Männedorf, Switzerland).

### 2.6. Glucose-Uptake Assay 

For this assay, 1.0 × 10^4^ cells were suspended in 200 μL culture media and treated with CAP or carrier gas argon for 10 s. Then, 800 μL of culture media were added and cells were incubated at 5% CO_2_ and 37 °C for 5 days. Glucose concentration in the cell-free supernatant was determined with an enzymatic glucose kit (R-Biopharm, Pfungstadt, Germany) after 4 h, 24 h, 48 h, 72 h, 96 h, and 120 h. Cells were harvested and counted with a CASY cell counter and analyzer. The relative glucose concentration per cell was calculated.

### 2.7. Live-Dead Assay

Cells were treated with CAP or argon for 10 s and were transferred to 96-well plates. Directly after treatment, after 24 h, 48 h, and 72 h, cells were stained with a live/dead cell imaging kit (Thermo Fisher Scientific, Waltham, MA, USA). Pictures were analyzed using ImageJ. Viable and dead cells were counted, and the fraction of dead cells was calculated.

### 2.8. Caspase-3/7 Assay

Cells were treated with CAP or argon for 10 s and were incubated for 48 h and 72 h (CAL-78) or 24 h and 48 h (SW1353). A second cell culture plate was treated identically to count cells. After the incubation period, the medium was replaced with 100 μL of caspase-3/7 detection solution (CellEvent™ caspase-3/7 green detection reagent (Thermo Fisher Scientific, Waltham, MA, USA), 2 µM in DPBS) and incubated for 45 min. The fluorescence was recorded at 535 nm using a microplate reader (Tecan m200, Männedorf, Switzerland).

### 2.9. TUNEL Assay

Cells were treated with 10 s CAP or carrier gas argon and were transferred to a 96-well plate. A second plate was plated in parallel, to normalize the measured fluorescence intensity to the cell number. TiterTACS Colorimetric Apoptosis Detection Kit (Trevigen, Gaithersburg, MD, USA) was performed according to the manufacturer’s instructions after 48 h and 72 h (CAL-78) or 24 h and 48 h (SW1353). Absorption was recorded using a microplate reader (Tecan m200, Männedorf, Switzerland).

### 2.10. Radius Cell Migration Assay

Cells were seeded 24 h before the start of the assay. The gel spot was removed according to the manufacturer’s instructions. Cells were washed with culture media, then 200 μL CAP or argon-treated medium was added. The assay was performed under low-serum conditions (CAL-78: 1% FCS, SW1353: 0.1% FCS). Cells were stained with staining solution after 24 h of incubation and imaged. Evaluation of the cell-free area was determined with ImageJ software, using the MRI wound-healing tool plug-in.

### 2.11. Hydrogen Peroxide Assay

For this assay, 200 μL of PBS was treated with CAP for 5, 10, 20, 40, and 80 s. We performed each examination with 3.0, 3.5, 4.0, 4.5, and 5.0 of SLM gas flow. The PBS was treated analogously to the cell suspensions in our other experiments. The treated PBS was diluted at 1:100 and an Amplex Red hydrogen peroxide/peroxidase assay (Thermo Fisher Scientific, Waltham, MA, USA) was carried out according to the manufacturer’s instructions. The data were analyzed using linear regression analysis.

### 2.12. Data Analysis

For data analysis and visualization, GraphPad Prism Version 9.1.2 (GraphPad Software Inc., La Jolla, CA, USA) was used. The results of *p* ≤ 0.05 of at least three independent experiments were considered significant and data were given as the mean ± SD. Differences were examined using a two-way ANOVA, followed by Sidak’s multiple comparison test. For experiments with repeated measurements at different times (e.g., the glucose uptake assay), repeated-measure two-way ANOVAs were used. The normal distribution of differences between groups was checked with the Shapiro–Wilk normality test.

## 3. Results

### 3.1. Cell Proliferation

**Figure 1:**
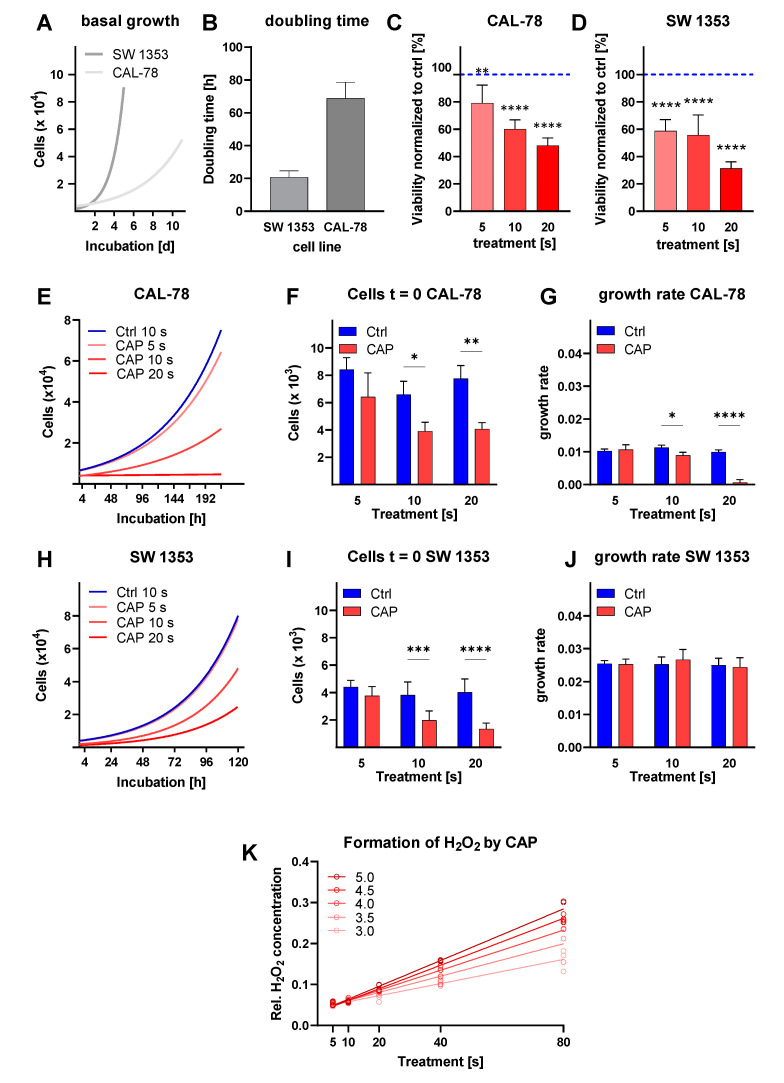
Chondrosarcoma (CS) cells were incubated for 5 d (SW 1353) and 11 d (CAL-78). Viable cells were counted daily. A growth model was calculated for each cell line (**A**) and the *doubling times* with mean ± SD were calculated (**B**). CellTiter-Blue cell metabolic activity assays were performed 72 h after treatment of CAL-78 (**C**) and SW 1353 with CAP (**D**). Data were given as mean ± SD viability compared to control. CS cells were treated with CAP and carrier gas argon for 5 s, 10 s, and 20 s, and incubated over 216 h (CAL-78) and 120 h (SW 1353). Cells were counted at the indicated time points, and growth models were calculated (**E**,**H**). The number of cells at t = 0 (**F**,**I**) and growth rate (**G**,**J**) were determined and shown as mean ± SD. PBS was exposed with different CAP treatment times combined with different gas flow rates, and the resulting concentration of H_2_O_2_ was measured (**K**). A linear increase in reactive species concentration due to increases in both gas flow rate and treatment time was demonstrated. Data were given as mean ± SD; significant differences are shown as follows: * *p* < 0.05, ** *p* < 0.01, *** *p* < 0.001, **** *p* < 0.0001.

To characterize the growth properties of the cell lines, we performed growth kinetics and analyzed them using a regression model. The cell lines showed a different growth behavior (Figure 1A,B). The CAL-78 cells grew slowly, dividing only every 68.7 h (95% CI: 60.3–78.6 h). In contrast, the mean *doubling time* of SW 1353 cells was 20.7 h (95% CI: 17.2–24.6 h).

After CAP treatment, CS cell metabolic activity was determined using the CellTiter Blue assay after 72 h of incubation. A reduction in metabolic activity was observed in both cell lines (Figure 1C,D). A 5-second CAP treatment of CAL-78 reduced the metabolic activity to 79.2 ± 13.2% (*p* = 0.0078). The effect was more pronounced after a 10-second treatment, which resulted in a reduction to 60.2 ± 6.7% (*p* < 0.0001), and a 20-second treatment, which reduced the metabolic activity to 48.0 ± 5.7% (*p* < 0.0001). Similar tendencies were also demonstrated by examining SW 1353. A 5-second treatment was sufficient to decrease the metabolic activity to 58.8 ± 8.3%. The growth-inhibiting effect was increased further in this cell line by extending the treatment period (*p* < 0.0001 in each case).

Once adjusted to the different growth behaviors, the kinetics for CAL-78 were carried out over 216 h (Figure 1E–G) and over 120 h for SW 1353 (Figure 1H–J). The immediate effect of CAP treatment by CAL-78 cells showed a significant reduction in cell numbers (*p* < 0.0001) compared to untreated (control) cells. The cell division rate was significantly reduced after CAP treatment (*p* < 0.0001). While the *doubling time* of CAL-78 in the control treatment was in the range of about 67 h (95% CI: 60.5–76.5 h) and, with a 5-second CAP treatment, showed no significant differences, the 10-second CAP treatment showed a prolongation of the *doubling time* to 77.6 h (95% CI: 63.9–98.8 h). The 20-second CAP treatment stopped cell division almost completely, as cells theoretically divided every 1040 h (95% CI: 288.6 h—infinity). In CAP-treated SW 1353, an immediate and significant reduction of cell count could be seen (*p* < 0.0001). There was no significant influence on this cell line’s growth rate (*p* = 0.8225). The *doubling time* of this cell line ranged around 27 h (95% CI: 25.2–37.7 h) independent of CAP treatment times.

In order to verify the concentration of H_2_O_2_ in liquids, caused by CAP treatment, this was measured after exposure for different CAP treatment times, combined with the different gas flow rates on the PBS, using an Amplex Red assay (Figure 1K). The concentration of the produced reactive species could be influenced by both the gas flow rate and the treatment time. The increase in H_2_O_2_ concentration increased linearly with increasing treatment time (R^2^ of at least 0.92). The slope of the curves of different gas flow rates differed significantly (*p* > 0.001).

### 3.2. Glucose Metabolism

**Figure 2:**
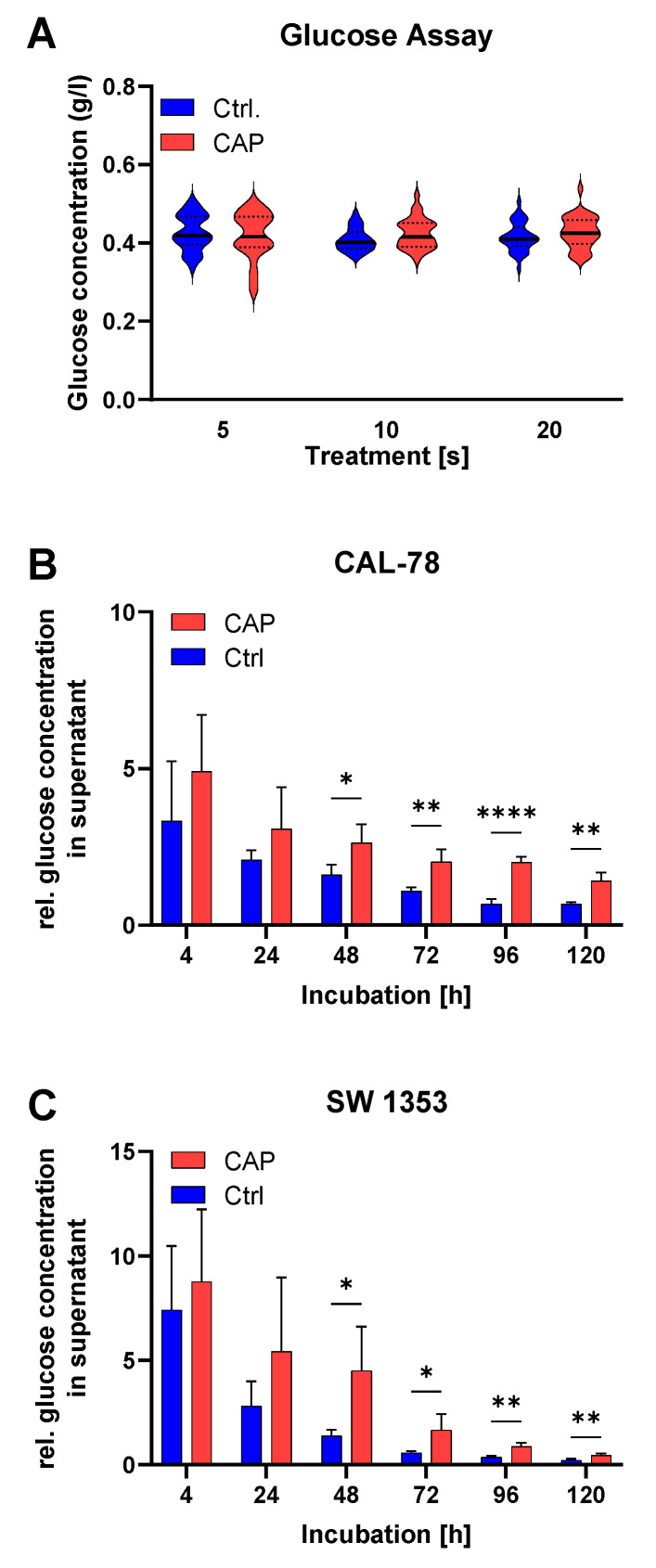
In the glucose assay, the concentration of D-glucose after CAP using different treatment times was measured (**A**). Glucose concentration in the cell-free supernatant was measured with an enzymatic glucose assay conducted 4 h, 24 h, 48 h, 72 h, 96 h, and 120 h after 10 s of CAP or control treatment (**B**,**C**). Glucose concentration was normalized to the cell count. Data were given as mean ± SD; significant differences are shown as follows: * *p* < 0.05, ** *p* < 0.01, **** *p* < 0.0001.

Various glucose experiments were carried out. To study the stability of D-glucose under the given conditions of CAP exposure, a glucose solution containing 400 mg/L was treated with CAP using different treatment times. It was found that the CAP treatment had no effect on the concentration of D-glucose in the aqueous solution, regardless of the exposure time (Figure 2A).

The measurement of the glucose concentration in the cell culture medium—once normalized to the respective cell numbers—showed a higher glucose concentration after CAP treatment (Figure 2B,C). This effect could be observed for both CAL-78 (*p* = 0.0057) and SW 1353 cell lines (*p* = 0.0299).

### 3.3. Apoptosis

**Figure 3:**
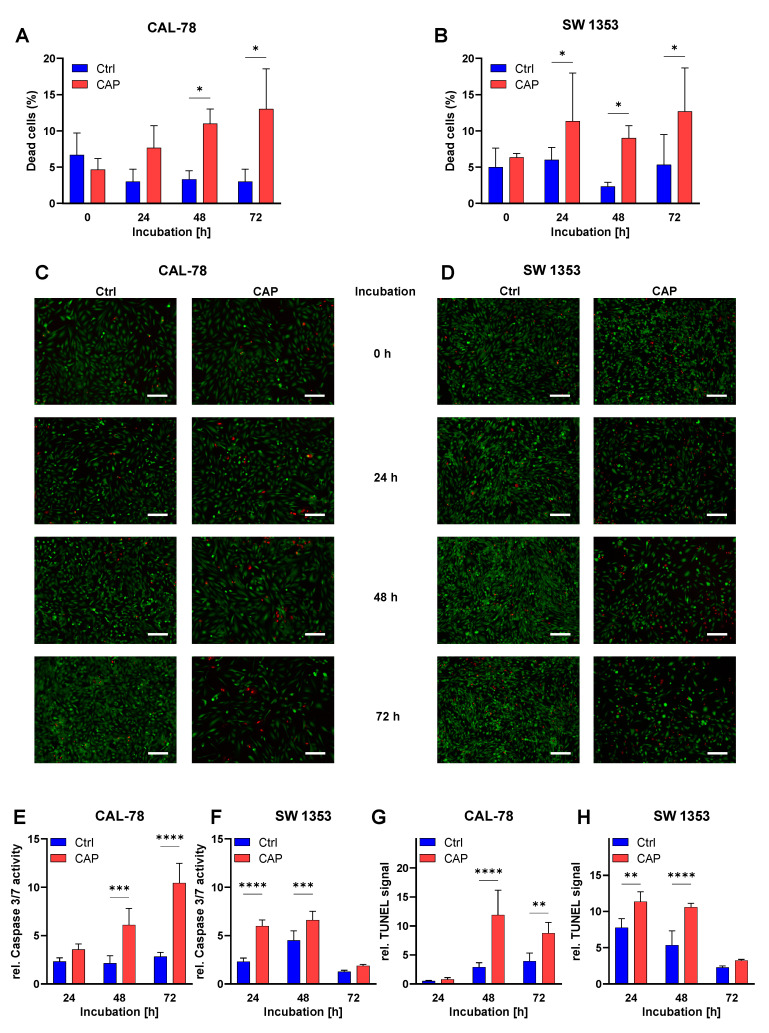
Chondrosarcoma (CS) cells were treated with CAP or carrier gas for 10 s. After 0 h, 24 h, 48 h, and 72 h of treatment, cells were stained with a Live/Dead cell imaging kit. Pictures were analyzed with ImageJ. Viable and dead cells were counted, and the fraction of dead cells was calculated (**A**,**B**). Data were given as mean ± SD. Representative images of stained CAL-78 (**C**) and SW 1353 (**D**) cells are shown for the apoptosis assays of caspase-3/7 (**E**,**F**) and TUNEL (**G**,**H**) at 24 h, 48 h, and 72 h (CAL-78), and 24 h, 48 h, and 72 h (SW 1353), with 10 s of CAP treatment. Data were given as mean ± SD of relative fluorescence (**E**,**F**) or absorption. Significant differences are indicated as follows: * *p* < 0.05, ** *p* < 0.01, *** *p* < 0.001, **** *p* < 0.0001. Scale bar = 200 µm.

Live-dead cell imaging was performed after CAP treatment. With the progress of the incubation time, the proportion of dead cells in both cell lines increased (Figure 3A–D). For CAL-78 cells, the differences between the CAP-treated and the control cells became significant 48 h after treatment (*p* < 0.0001). This difference was significant in SW 1353 cells as early as 24 h after treatment (*p* = 0.0288).

After the CAP treatments were performed, two different assays for apoptosis detection were employed. The assays in these experiments were also performed at cell-line-specific incubation time points, in order to adapt to the growth characteristics of the individual cell lines (Figure 3E–H). After 24 h, there was no significant increase in the apoptotic signals in CAL-78 cells. However, there was a significant increase after 48 h and 72 h (TUNEL 48 h: *p* < 0.0001, 72 h: *p* = 0.0128, caspase-3/7: 48 h: *p* = 0.0169, 72 h: *p* = 0.0003) and after 24 and 48 h in SW 1353 (TUNEL 24 h: *p* = 0.0243, 48 h: *p* = 0.0031, caspase-3/7: 24 h: *p* = 0.0007, 48 h: *p* = 0.0188). However, after 72 h, this cell line showed no significant increase in apoptosis signals.

**Figure 4:**
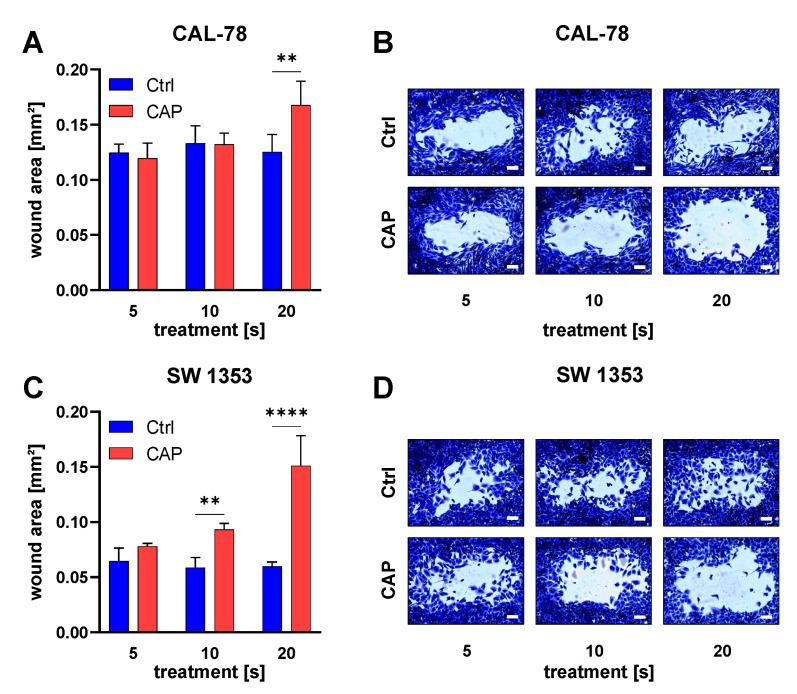
Cell migration shown 24 h after exposure to CAP-treated cell culture medium. The cell-free area was measured with ImageJ (**A**,**C**). Representative selection of images after incubation for 24 h for both cell lines with different CAP-treatment times (**B**,**D**). Significant differences are indicated as follows: ** *p* < 0.01, **** p < 0.0001. Scale bar = 200 µm.

The migration assay showed that CAP treatment inhibited the motility of chondrosarcoma cells (Figure 4). This effect was related to the duration of CAP exposure of the culture medium. A longer exposure time was associated with a greater reduction in cell motility. The effect was more pronounced in the SW cell line. At the end of the incubation period, the cells treated with CAP for 20 s showed a non-invaded area that was more than twice as large as in the controls (*p* < 0.0001). A 20-second treatment of the culture media of CAL-78 cells also leads to a significantly larger non-invasion area after incubation (*p* = 0.0680).

## 4. Discussion

With several approved devices on the market and CAP-supported wound healing being performed in dozens of clinical centers in central Europe, the application of plasmas has entered clinical practice [20]. In recent years, this has been extended to the field of experimental oncology [21,22].

It is known that cancer cells generally have lower cholesterol levels in the cell membrane and are, therefore, more susceptible to peroxidation [23,24]. This membrane lipid peroxidation leads to an increase in the porosity of the cell membrane and also to an increase in the diffusion of reactive oxygen and nitrogen species (*RONS*) into the tumor cell [24]. One of CAP’s primary mechanisms of action is attributed to the production of RONS. Our current experiments with the kINPen Med confirm the production of *RONS*, specifically in this context, the formation of hydrogen peroxide by CAP. This production shows a linear increase depending on the CAP exposure time. Other studies also confirm the linear relationship between *RONS* development and CAP treatment time [25,26]. We observed cell membrane changes in our first study of the effects of CAP on CS cells [18] and recently confirmed the correlation between cholesterol content and CAP-induced cytotoxicity in tumor cells [27]. Moreover, studies have shown that the *RONS* in CAP cause changes in the antioxidant system and oxidative stress in cancer cells [28,29]. In a review, Brany et al. consider CAP application as a possible replacement for radiation, although possible DNA damage to the cells is an undesirable effect [30]. Other studies also report DNA damage caused by CAP treatment, wherein VU radiation and *ROSN* were included as consequences [31,32,33,34]. However, other authors postulate that DNA damage is not a direct consequence of CAP treatment [35,36]. CAP has shown stimulative potential across several non-malignant cell types [37,38]. However, a genuinely selective effect on cancer cells is unproven [27] but is also less important since most local therapies, such as cryo-ablation, laser treatment, and surgery, are less selective. In tumor cells, CAP was shown to induce apoptosis, growth inhibition, and mitochondrial damage, to name a few consequences [22].

CAP application in primary osseous tumors, such as osteosarcoma and Ewing’s sarcoma, was specifically examined in the context of in vitro studies. Above all, the antiproliferative effects of CAP and the triggering of apoptosis mechanisms have been demonstrated [17]. This study investigates the CAP treatment effect on another skeletal tumor—CS. Using two different cell lines of CS with different growth properties, growth kinetics were carried out and analyzed using a regression model to adapt the growth behavior of the cell lines for further experiments. After CAP treatment, a reduction in cell viability was observed in both cell lines. This CAP effect was treatment-time-dependent and its impact increased with the longer exposure time. Such a treatment-time-dependent effect of CAP has been described in several studies and also appears to be independent of the CAP source. Antiproliferative effects after CAP exposure were observed for both cell lines. The growth kinetics of CAL-78 cells after CAP treatment suggests two effects. On the one hand, significantly more cells were killed by CAP treatment than in the control with carrier gas argon. On the other hand, the cell division rate was significantly reduced by CAP-treated cells. This could be explained by alterations of the cell cycle [39,40] and the observed reduced metabolism. An immediate significant reduction in the cell count of the CAP-treated cells was shown for both cell lines. The effect of immediate reduction after exposure to CAP was also observed in osteosarcoma cell lines [41].

The measurement of glucose concentration in the cell culture medium showed increasing glucose concentration after CAP exposure for both cell lines. In order to rule out the possibility that the glucose might be decomposed by the CAP treatment, in the current study, glucose solution was exposed to CAP. It was shown that the glucose remains unaffected, even after longer CAP treatment times. We conclude from the lower glucose turnover that there is a reduced metabolism after CAP treatment. Similar CAP effects on the cells’ glucose metabolism have also been described in the human endothelial cell line [42]. Other studies on this topic also indicate that glucose uptake and metabolism are affected by CAP treatment [43,44].

Furthermore, we measured the proportion of dead cells in both cell lines, using live-dead cell imaging after CAP treatment. Looking at the time course of the cell incubation, a significant difference in the ratio of dead/alive cells compared to the control group was already detected after 24 h for the SW 1353 cell line and for the CAL-78 cells after 48 h. To understand the mechanisms behind this CAP effect, we looked for apoptotic processes in the CS lines, as these were also found in other tumor cells [45,46]. Performing two different apoptosis assays, we found a significant increase in apoptotic signaling in both CS cell lines after CAP exposure. Thus, the effects of CAP treatment on CS cells approximate the CAP effects on other skeletal tumor cells, such as osteosarcoma and Ewing’s sarcoma, and appear to be due to cell growth inhibition and apoptotic processes [17,47,48,49]. Due to the oxidative stress of CAP treatment on the cells, other cell-death processes such as necrosis are also conceivable, in addition to the apoptosis processes. Our own preparatory work on the effects of CAP treatment on CS showed a significant impact on the cell membrane of CS cells [18]. The damaged integrity is certainly connected with the necrotic processes of the cell [50]. Other authors also observed necrotic processes in tumor cells, which are mainly attributable to the physical factors of the CAP treatment [51]. In the current work, the reduction in cell number immediately after CAP treatment may be an indication of the necrotic elimination of the cells. However, the results of the live-dead assay directly after CAP treatment show no significant changes, compared to the control group. Thus, we assume that even if necrotic processes take place after CAP treatment, these do not represent its central and decisive role.

The inhibition of cell motility by CAP treatment or treatment with CAP-activated fluids was described for serval tumor entities [52], for example, ovarian or breast cancer [53,54] and multiple myeloma cells [55]. This could be due to changes in the actin cytoskeleton and the decreased secretion of matrix metallopeptidases [39,55]. The study presented here describes for the first time the effect of CAP treatment on the motility of CS cells. Here we observed in the Radius assay that indirect treatment of CS cells with CAP causes significant inhibition of their motility. This is another important cell function that is noticeably inhibited in CS cells, depending on the duration of CAP treatment. Cell mobility is certainly a parameter that can also be influenced depending on the experiment. In the current experiments, however, the conditions for both the CAP-treated and the control (untreated) cancer cells were kept the same, so that a statement on the parameter cell motility was possible. This is an important finding for assessing the impact of CAP treatment on CS cells, as it also allows statements to be made about the important life functions of the cancer cells.

The current study presents for the first time the effects of CAP treatment on cell metabolism, motility, and growth, as well as apoptotic processes in chondrosarcoma. The in vitro results indicate that CAP could become a promising anticancer tool for such rare but difficult-to-treat bone tumors as CS. This work is essential for further steps toward in vitro and clinical applications in chondrosarcoma. A conceivable outlook for the future may be that direct CAP therapy is used during the surgical resection of the tumor. Thus, tumor remnants after resection could also be addressed for possible incomplete resections, potential incomplete resections, or potential tumor recurrences. Options for therapy with CAP include direct CAP application to the surgically resected wound surface or intraoperative rinsing with CAP-activated liquids as an indirect CAP treatment. Both methods appear to be feasible for intraoperative use; however, the impact on a CS tumor mass in vivo needs to be studied before a clinical application is conceivable.

## Data Availability

Data are available from the corresponding author upon reasonable request.
